# Depression in dialysis patients with end-stage kidney disease: investigating the role of psychosocial stressors, co-occurring medical conditions and demographic influences

**DOI:** 10.1192/bjo.2026.11039

**Published:** 2026-05-11

**Authors:** Kamaldeep Bhui, Christophe Clesse, Simone Rahman, Georgina Mayling Hosang, Brian Gracey, Fiona Loud, Simon Kirwin, David Randall, Karl Marlowe, Livia A. Carvalho, Magdi M. Yaqoob

**Affiliations:** Department of Psychiatry and Wadham College, University of Oxford, UK; Oxford Health NIHR BRC, Oxford, UK; East London NHS Foundation Trust, London, UK; School of Psychology, Roehampton University, UK; Centre for Psychiatry and Mental Health, Wolfson Institute of of Population Health, Queen Mary University of London, UK; Renal Patient, Barts Health NHS Trust, London, UK; Renal Medicine, Barts Health NHS Trust, London, UK; Kidney Care UK, Alton, UK; Board Executive, Oxford Health NHS Foundation Trust, Oxford, UK; Clinical Pharmacology and Precision Medicine, Queen Mary University of London, UK; William Harvey Institute, Queen Mary University of London, UK

**Keywords:** Psychological medicine, dialysis, depression, psychosocial adversity, sex and ethnicity

## Abstract

**Background:**

Depression is common in end-stage kidney disease (ESKD) and is associated with poorer outcomes and higher mortality. However, treatment guidance is inconsistently applied.

**Aims:**

To investigate screening measures, somatic symptoms, comorbidities and psychosocial and cultural influences on depression diagnosis in ESKD patients.

**Method:**

We recruited 300 people with ESKD receiving maintenance hospital haemodialysis in a deprived ethnically diverse area. We assessed depression using validated screening tools (Hamilton Depression Rating Scale and Patient Health Questionnaire 2) and a definitive ICD-10 diagnosis using a structured interview (Clinical Interview Schedule-Revised). We considered the role of adverse life events, co-occurring medical conditions, as well as age, sex and ethnicity, using descriptive statistics and multiple logistic regression.

**Results:**

An ICD-10 diagnosis of *moderate or severe depression* was made in 8% of the sample, taking care to exclude potentially confounding symptoms associated with chronic kidney disease and depression. Applying validated thresholds on commonly used screening tools yielded substantially higher prevalence estimates. An ICD-10 diagnosis of moderate and severe depression associated with loss events: death of a spouse, child or parent (odds ratio 3.62, 95% CI: 1.09–12, *p* = 0.04), financial strain (odds ratio 3.51, 95% CI: 1.04–11.87, *p* = 0.04), type 2 diabetes (odds ratio 5.32, 95% CI: 1.34–20.76, *p* = 0.02) and education, whereby university graduates were less likely to have depression than school-only attendees (odds ratio 0.18, 95% CI: 0.03–1.02, *p* = 0.05). Ethnicity and sex were not significantly associated with moderate or severe depression.

**Conclusions:**

We found a lower prevalence of moderate to severe depression than commonly reported. Future research should consider careful diagnostic assessment, financial strain, loss events and physical co-occurring medical conditions such as diabetes.

Depression is common in chronic medical conditions and is associated with poorer quality of life, greater mortality, and increased service use and care costs.^1,2^ Depression has an estimated global prevalence of around 26 to 39% in chronic kidney disease (CKD),^3^ and is associated with increased mortality among patients receiving haemodialysis.^4^ However, depression is often under-recognised and under-treated in dialysis patients.^5,6^ The implementation of evidence-based guidance to assess and treat depression for those on dialysis is inconsistently applied.^7^ There are many potential explanations for this:

- Somatic symptoms are common in depression and in chronic medical conditions, so evaluating their diagnostic significance requires care.
- CKD is associated with advancing age and comorbidities including hypertension, cardiovascular disease and diabetes.^8^ Each of these adds to the risks of depression, and can create diagnostic uncertainty.
- The adverse effects associated with pharmacotherapy may deter their use despite evidence of benefits.^9–14^
- In routine care, a diagnosis is usually made by clinical assessment or through screening measures, rather than detailed structured assessments. Each approach has strengths and weaknesses.^15^ Furthermore, liaison psychology or psychiatry services offering detailed assessment may not be accessible to all.
- Diagnostic uncertainty can arise from the complex interplay of social and psychological factors. Depression is more common among people experiencing adversity, traumatic life events and discrimination, aligned more with *eco-social* rather than *individual biomedical models* of illness.^16–19^ Social adversities and inequalities are also common in people undergoing dialysis.^5,20–22^
- When assessing ethnically diverse populations, there is a greater chance of under-estimating levels of depression. For example, among South Asians the symptoms of depression may be influenced by idioms of distress, literacy and cultural expectations; depressive symptoms often present in less intense ways.^23^ Some research suggests screening measures operate differently across ethnic and cultural groups.^24–26^ Consequently, comparison of assessment methods may help guide clinicians in careful diagnosis.

There is evidence of poorer outcomes of ESKD by race or ethnicity, although this evidence is inconsistent and contested.^27,28^ At the same time, minority ethnic groups and those living in precarity are known to be under-represented in research generally and in research on chronic medical conditions,^29,30^ leading to calls for more research on ethnicity, co-occurring medical conditions and an array of psychosocial influences.^31^

This research aims to:

- estimate the prevalence of ICD-10 diagnoses of *moderate to severe depression* in ethnically diverse patients with end-stage kidney disease receiving dialysis in a large, urban haemodialysis service;
- compare the estimates of depression on routinely used and validated screening instruments with an ICD-10 diagnosis of *moderate to severe depression*;
- consider eco-social models of illness, and test relationships between psychosocial adversities (life events and discrimination), co-occurring medical conditions, demographics (age, sex and ethnicity) and respective risks of moderate to severe depression.

## Method

Barts Health provides care for people of diverse ethnic backgrounds in one of the most deprived parts of the country. The renal service at Barts Health NHS Trust runs in haemodialysis units at hospitals in Northeast London and Essex in the UK (including the Royal London Hospital, Newham General Hospital, King George’s Hospital, Queens Hospital and Whipps Cross Hospital), as well as a large home haemodialysis service.

After sharing information about the study, and seeking advice about suitability from their clinicians, patients providing *written informed* consent were interviewed and their clinical records were reviewed. Patients were recruited while attending dialysis and were free to withdraw from the study at any point. Included patients were attending in-patient dialysis and approved to participate by clinicians following preliminary discussion with the patient to indicate willingness, ability to participate and judged to have capacity.

Three hundred patients receiving maintenance haemodialysis under the care of Barts Health NHS Trust were interviewed between October 2021 and September 2022. This was a convenience sample of those admitted for dialysis. We identified that 62 patients refused and 22 did not meet inclusion criteria (severe illness, frailty, marked language difficulties). Overall, this gives us a response rate of 82.8% of eligible patients across centres (see Supplementary material A (available at https://doi.org/10.1192/bjo.2026.11039) for response rates by recruitment centre).

The authors assert that all procedures contributing to this work comply with the ethical standards of the relevant national and institutional committees on human experimentation and with the Helsinki Declaration of 1975, as revised in 2013. All procedures involving human subjects/patients were approved by Health Care Research Wales (reference 19/LO/1272; IRAS project ID 256696). Queen Mary University of London was the sponsor.

### Interview data

In the interview, we asked about demographics, social adversities and mental health status. Additional clinical data were extracted from care records.

#### Demographics

The interview questions include age, sex, ethnicity (White British, South Asian, Black including Black British, Black African, Black Caribbean and Other), occupation (unemployed, employed, retired, student, working as housewife, off sick from work, other) and education (none, primary and secondary school attendance with or without completion, degree or apprenticeship level or higher, or Other meaning any other courses not formally recognised in the aforementioned categories that may be relevant to education in different countries). We also asked about marital status and smoking and alcohol use.

#### Discrimination and life events

Experiences of discrimination in the previous 12 months were assessed using 5 questions from the EMPIRIC study, including experience of unfair treatment at work, job refusal, physical attack, property damage or racial insults.^18^ A 12-item measure of threatening life events in the previous 12 months was completed. Subjects were asked if they had experienced serious illness, injury or attack; a close relative suffering serious illness, injury or attack; the death of a parent, child or spouse; the death of a close family friend; the end of a significant relationship; serious problems with neighbours, a close friend or relative; unemployment or seeking work unsuccessfully for a month; loss of work; a major financial crisis; problems with the police or courts; or loss or damage to something they valued.^32^

#### Depression measures

We evaluated the 2-item version of the Patient Health Questionnaire (PHQ2),^33^ the 17-item version of the Hamilton Depression Rating Scale (HAMD)^34^ and the depression items of the Clinical Interview Schedule-Revised (CIS-R)^35^ required for classification of ICD-10 diagnoses. We used an algorithm for the CIS-R items to make ICD-10 diagnoses of depression (see Supplementary material B): no depression, mild depression, combined moderate and severe depression. The algorithm to generate ICD-10 diagnoses was that used in the EMPIRIC study^36^ and the Office of National Statistics’ Adult Psychiatric Morbidity Survey (2014),^37^ with re-routing of questions to avoid repetition of items and to minimise questionnaire length. We combined diagnostic groups with or without somatic symptoms (the breakdown by sex is available in the Supplementary material C). Of note, some of the appetite, weight and social impairment symptoms items of the CIS-R were felt to be so common to all dialysis patients when piloting the questionnaires, so to avoid diagnostic confusion, these items were not asked or scored. These items, we felt, were inappropriate as nutrition and appetite are affected by dialysis, inflammation, or depression.^38^ We did not ask the impairment question, as all dialysis in-patients’ dialysis were impaired in daily function and routine. This also meant we were able to balance the items needed for a confident diagnosis of depression versus demands made of frail patients undergoing dialysis at the time of the interview.

The interview was undertaken by an experienced post-doctoral clinical psychologist aware of the common symptoms and presentations of depression (C.C.). Derived diagnoses of depression were aligned with C.C.’s clinical judgement. We also assessed depressive symptoms based on conventional thresholds for common validated screening instruments that are used in routine clinical practice and research (PHQ2, threshold of 3 or more; threshold HAMD, threshold of 19 or more). Specifically, the PHQ2 was already in use in the clinical services as a short and practical measure of depression to alert clinicians to consider depression support.

Clinical data gathered from electronic records included type of dialysis, comorbidities (hypertension, diabetes types 1 and 2, obesity, cardiovascular disease, current or past cancer, obesity, chronic obstructive airways disease, autoimmune disorders) and a previous record of seeing a specialist for mood disorder.

### Data management and statistical analysis

Data were extracted from clinical records, linked to patient interviews, anonymised, stored in Excel and analysed in Stata version 16.1 (by K.B.; https://www.stata.com). The ICD-10 codes were generated in R version 4.3.3 (by C.C.; https://posit.co/download/rstudio-desktop; see Supplementary material B for algorithms and code).

### Cohort description by overall and by sex and ethnic group

First, we examined demographic characteristics, social variables such as discrimination and life events overall and then by sex. We classified depression as ICD-10 diagnoses of *mild depression* and of *moderate or severe depression*, and by binary measures of depression on screening instruments (PHQ2 score ≥ 3; and HAMD score ≥ 19). We assessed demographics by depression and long-term medical conditions, overall and by sex. We then examined the same variables by ethnic groups.

### Statistical associations with ICD-10 diagnosis of moderate or severe depression

The approach to statistical analysis was framed within eco-social and syndemic models of illness, whereby many contextual and individual variables can interact. Therefore, we undertook data reduction to restrict more complex models to relevant variables of sufficient quality.

In cross-tabulations, we applied chi-squared statistics and Fisher’s Exact test where cell values were less than 5. We ran separate stepwise backward logistic regression models for the following three blocks of variables, retaining those that were associated with depression at *p* < 0.1 level, for a final fully adjusted model.

- Block 1: Demographics: age, sex, marital status, education, occupation, alcohol and smoking.
- Block 2: Psychosocial variables: life events and discrimination items.
- Block 3: Long-term conditions (hypertension, diabetes, chronic obstructive pulmonary disease, cardiovascular disease, obesity, current or past cancer, autoimmune disorders).

Variables associated with depression at *p* < 0.1 were entered in a full regression model, assessed for model fit by *R* ^2^ value, and noting significant variables if associated at *p* < 0.05 level.

## Results

### Cohort description by sex and ethnicity

The sample’s characteristics are shown overall and by sex (Table 1), and then depression and co-occurring medical conditions are shown overall and by sex (Tables 1 and 2). The same variables are then shown by ethnicity (Tables 3 and 4). Men were more likely to be married, employed, current or past smokers and consumers of alcohol than women. Women were more likely to have adverse life events than men. The South Asian and Black groups were younger than the White British group. The White British group was more likely to smoke and drink alcohol. The Black and South Asian groups were more likely to have experienced discrimination. Black people were more likely to be single. South Asians were most likely to be married.

**Table 1:** Demographics and social characteristics: overall and by sex

Demographics		Total, *n* (%)	Male (177), *n* (%)	Female (123), *n* (%)	*X* ^2^	d.f.	*p*
Age							
≤40		33 (11)	14 (7.9)	19 (15.5)	4.21	2	0.121
41–60		106 (35.3)	65 (36.7)	41 (33.3)			
≥61		161 (53.7)	98 (53.4)	63 (51.2)			
Marital status							
Single		81 (27)	49 (27.7)	32 (26.0)	6.15	2	0.046
In relationship or married		161 (53.7)	102 (57.6)	59 (48)			
Divorced or widowed		58 (19.3)	26 (14.7)	32 (26)			
Highest education							
None		76 (25.3)	43 (24.3)	33 (26.8)	1.28	3	0.734
School		133 (44.3)	77 (43.5)	56 (45.5)			
Degree or apprenticeship		77 (25.7)	47 (26.6)	30 (24.4)			
Other/missing		14 (4.67)	10 (5.7)	4 (3.3)			
Occupation							
Unemployed		55 (18.4)	33 (18.6)	22 (17.9)	13.74	6	0.033
Employed		56 (18.7)	36 (20.5)	20 (16.3)			
Retired		109 (36.5)	65 (36.9)	44 (35.8)			
Student		2 (0.67)	1 (0.6)	1 (0.6)			
Housewife		8 (2.7)	0	8 (6.5)			
Sick		1 (0.3)	0	1 (0.8)			
Missing		68 (22.7)	41 (23.3)	27 (22.0)			
Ethnicity							
White		91 (30.3)	60 (33.9)	31 (25.2)	3.96	3	0.265
Asian		101 (33.7)	57 (32.2)	44 (35.8)			
Black		100 (33.3)	54 (30.5)	46 (37.4)			
Other		8 (2.7)	6 (3.4)	2 (1.6)			
Smoker							
Yes		33 (11)	22 (12.4)	11 (8.9)	9.93	2	0.007
No, used to		68 (22.7)	50 (28.3)	18 (14.6)			
No, never		199 (66.3)	105 (59.3)	94 (76.4)			
Alcohol use							
Never		209 (69.7)	111 (62.7)	98 (79.7)	9.93	2	0.007
Occasionally		88 (29.3)	64 (36.2)	24 (19.5)			
At least once a day		3 (1)	2 (1.1)	1 (0.8)			
Discrimination (any events)							
No		255 (85)	152 (85.9)	103 (83.7)	0.26	1	0.61
Yes		45 (15)	25 (14.1)	20 (16.3)			
Life events (number of items endorsed)							
None		42 (14)	22 (12.4)	20 (16.3)	11.25	2	0.004
Once		137 (45.7)	95 (53.7)	42 (34.2)			
2 or more		121 (40.3)	60 (33.9)	61 (49.6)			

**Table 2:** Health: overall and by sex

Condition		Total, *n* (%)	Male, *n* (%)	Female, *n* (%)	*X* ^2^	d.f.	*p*
Overall mild depression (F3200 + F3201)							
No		247 (82.3)	146 (82.5)	101 (82.1)	0.006	1	0.94
Yes		53 (17.7)	31 (17.5)	22 (17.9)			
Overall depression moderate + severe (F32.01 + F32.10 + F32.11 + F32.22)							
No		276 (92)	167 (94.4)	109 (88.6)	3.24	1	0.07
Yes		24 (8)	10 (5.7)	14 (11.4)			
Hamilton							
0–18		216 (72)	137 (77.4)	79 (64.2)	6.25	1	0.01
19 or more		84 (28)	40 (22.6)	44 (35.8)			
PHQ2							
Score <3		229 (76.3)	141 (79.7)	88 (71.5)	2.65	1	0.1
Score 3 or more		71 (23.7)	36 (20.3)	35 (28.5)			
Known past depression							
No		291 (97)	171 (96.6)	120 (97.6)	0.225	1	0.64
Yes		9 (3)	6 (3.4)	3 (2.4)			
Type 1 diabetes							
No		275 (97.2)	161 (97.6)	114 (96.6)	0.233	1	0.63
Yes		8 (2.8)	4 (2.42)	4 (3.4)			
Type 2 diabetes							
No		170 (60.1)	97 (54.8)	73 (59.4)	0.272	1	0.6
Yes		113 (39.9)	68 (54.8)	45 (38.1))			
Hypertension							
No		125 (41.8)	75 (42.6)	50 (40.7)	0.115	1	0.44
Yes		174 (58.2)	101 (57.4)	73 (59.4)			
Cardiovascular disease							
No		184 (61.5)	99 (56.3)	85 (69.1)	5.05	1	0.03
Yes		115 (38.5)	77 (43.8)	38 (30.9)			
Chronic obstructive pulmonary disease							
No		291 (97.3)	170 (96.6)	121 (98.4)	0.885	1	0.35
Yes		8 (2.7)	6 (3.4)	2 (1.6)			
Past or present cancer							
No		263 (88)	153 (86.9)	110 (89.4)	0.427	1	0.51
Yes		36 (12)	23 ((13.1)	13 (10.6)			
Autoimmune/HiV							
No		254 (84.9)	155 ((88.1)	99 (80.49)	3.254	1	0.07
Yes		45 (15.1)	21 (11.9)	24 (19.5)			
Obesity							
No		277 (92.6)	167 (94.9)	110 (89.4)	3.161	1	0.08
Yes		22 (7.4)	9 (5.1)	13 (10.6)			
Multimorbidity (no of conditions) (including cardiovascular disease, hypertension, obesity, cancer, autoimmune/HiV)							
0		53 (17.7)	32 (18.2)	21 (17.1)	3.092	4	0.54
1		133 (44.5)	75 (42.6)	58 (47.2)			
2		96 (32.1)	56 (31.8)	40 (32.5)			
3		15 (5)	11 (8.3)	4 (3.3)			
4		2 (0.7)	2 (1.1)	0			

PHQ2, Patient Health Questionnaire.

**Table 3:** Demographic and social conditions by ethnicity

Demographics	White (*n* = 91), *n* (%)	Asian (*n* = 101), *n* (%)	Black (*n* = 100), *n* (%)	Other (*n* = 8), *n* (%)	*X* ^2^	d.f.	*p*
Age							
≤40	5 (5.5)	16 (15.8)	11 (11)	1 (12.5)	14.967	6	0.02
41–60	25 (27.5)	32 (31.7)	45 (45)	4 (50)			
≥61	61 (67)	53 (52.5)	44 (44)	3 (37.5)			
Marital status							
Single	25 (27.5)	16 (15.8)	40 (40)	0	30.488	6	<0.001
In relationship/married	41 (45.1)	70 (69.3)	42 (42)	8 (100)			
Divorced or widowed	25 (27.4)	15 (14.9)	18 (18)	0			
Highest education							
None	26 (28.6)	27 (26.7)	22 (22)	1 (12.5)	4.287	9	0.89
School	39 (42.9)	44 (43.6)	46 (46)	4 (50)			
Degree or apprenticeship	24 (26.4)	25 (24.8)	26 (26)	2 (25)			
Other/missing	2 (2.2)	5 (5.0)	6 (6)	1 (12.5)			
Occupation							
Unemployed	16 (17.6)	19 (19)	18 (18)	2	21.454	18	0.26
Employed	17 (18.7)	17 (17)	20 (20)	2			
Retired	41 (45.1)	38 ((28)	29 (29)	1			
Student	0	0	2 (2)	0			
Housewife	1 (1.1)	6 (6)	1 (1)	0			
Sick	0	0	1 (1)	0			
Missing	16 (17.6)	20 (20)	29 (29)	3			
Smoker							
Yes	15 (16.5)	8 (7.9)	8 (8)	2 (25)	19.055	6	<0.01
No, used to	31 (34.1)	19 (18.8)	17 (17)	1 (12.5)			
No, never	45 (49.5)	74 (73.3)	75 (75)	5 (62.5)			
Alcohol use							
Never	51 (56)	90 (89.1)	61 (61)	7 (87.5)	31.577	6	<0.001
Occasionally	38 (41.8)	11 (10.9)	38 (38)	1 (12.5)			
At least once a day	2 (2.2)	0	1 (1)	0			
Discrimination (any events)							
No	85 (93.4)	83 (82.2)	80 (80)	7 (87.5)	7.675		0.05
Yes	6 (6.6)	18 (17.8)	29 (20)	1 (12.5)			
Life events (number of items endorsed)							
None	6 (6.6)	17 (16.8)	18 (18)	1 (12.5)	12.385	3	0.05
Once	51 (56)	46 (45.5)	35 (35)	5 (62.5)			
2 or more	34 (37.4)	38 (37.6)	47 (47)	2 (25)			

**Table 4:** Health by ethnicity

Condition	White (*n* = 91), *n* (%)	Asian (*n* = 101), *n* (%)	Black (*n* = 100), *n* (%)	Other (*n* = 8), *n* (%)	*X* ^2^	d.f.	*p*
ICD-10							
No	84 (92.3)	93 (92.1)	92 (92)	7 (87.5)	0.233	3	0.97
Moderate or severe							
Yes	7 (7.69)	8 (7.92)	8 (8)	1 (12.5)			
Hamilton19	67 (73.6)	69 (68.3)	75 (75)	5 (62.5)	1.604	3	0.66
Hamilton							
No	19 (20.9)	20 (19.8)	29 (29)	1 (12.5)	18.083	12	0.11
Mild	38 (41.8)	26 (25.7)	32 (32)	4 (50)			
Moderate	10 (11)	23 (22.8)	14 (14)	0			
Severe	16 (17.6)	15 (22.8)	10 (10)	1 (12.5)			
Very severe	8 (8.8)	17 (16.8)	15 (15)	2 (25)			
PHQ2							
Score <3	68 (74.3)	78 (77.2)	77 (77)	6 (75)	0.208	3	0.98
Score 3 or more	23 (25.3)	23 (22.8)	23 (23)	2 (25)			
Known past depression							
No	88 (96.7)	96 (95)	99 (99)	0	2.97	3	0.4
Yes	3 (3.3)	5 (4.95)	1 (1)	0			
Type 1 diabetes							
No	86 (95.6)	87 (97.8)	95 (99)	7 (87.5)	4.805	3	0.19
Yes	4 (4.4)	2 (2.3)	1 (1)	1 (12.5)			
Type 2 diabetes							
No	54 (59.3)	12 (11.9)	4 (4)	0	16.847	6	0.01
Yes	36 (39.6)	43 (42.6)	32 (32)	2 (25)			
Missing	1 (1.1)	12 (11.9)	4 (4)	0			
Hypertension (HT)							
No	45 ((50)	49 (48.5)	28 (28)	3 (37.5)	12.248	3	<0.01
Yes	45 (50)	52 (51.5)	72 (72)	5 (52.5)			
Cardiovascular disease (CVD)							
No	55 (61.1)	56 (55.5)	68 (68)	5 (62.5)	3.358	3	0.34
Yes	35 (38.9)	45 (44.6)	32 (32)	3 (37.5)			
Chronic obstructive pulmonary disease							
No	87 (96.7)	98 (97.0)	99 (99)	7 (87.5)	4.227	3	0.24
Yes	3 (3.3)	3 (3.0)	1 (1)	1 (12.5)			
Past or present cancer (Ca)							
No	72 (80)	93 (92)	90 (90)	8 (100)	8.491	3	0.04
Yes	18 (20)	8 (7.9)	10 (10)	0			
Autoimmune/HiV (AI)							
No	85 (94.4)	83 (82.2)	80 (80)	6 (75)	9.489	3	0.02
Yes	5 (5.6)	18 (17.8)	20 (20)	2 (25)			
Obesity (Ob)							
No	82 (91.1)	96 (95.1)	91 (91)	8 (100)	2.199	3	0.53
Yes	9 (8.9)	5 (5)	9 (9)	0			
Multimorbidity (no of additional conditions) (including CVD, HT, Ob, Ca, AI)							
0	22 (24.4)	19 (18.1)	10 (10)	2 (25)	10.761	12	0.55
1	37 (41.1)	44 (43.6)	50 (50)	2 (25)			
2	25 (27.8)	33 (32.7)	35 (35)	3 (37.5)			
3	5 (5.6)	4 (4.0)	5 (5.0)	1 (12.5)			
4	1 (1.1)	1 (1.0)	0	0			

PHQ2, Patient Health Questionnaire.

### Health status

#### Overall and by sex (Table 2)

The ICD-10 diagnoses of depression reported included mild depression (*n* = 53) and moderate or severe depression (*n* = 24). The overall level of moderate or more severe depression was 8% (95% CI: 5.2–11.7; *n*/*N* = 24/300). Only 9 of the 300 (3%, 95 CI 1.4–3.6) had a history of seeking help for depression noted in the electronic records. There was a non-significant trend by sex, with women having a higher prevalence of moderate or severe depression (11.4 *v*. 5.7%). This seemed to be accounted for mainly by moderate or severe depression with somatic symptoms, see Supplementary material C).

Looking at the screening instruments for depression, on the HAMD, 22.6% of men and 35.8% of women scored above the moderate or more severe threshold (scoring 19 or above); on the PHQ2, 20.3% of men and 28.5% of women scored above the threshold score of 3 or more. Women were more likely to be classified as having very severe depression symptoms when assessed by the HAMD (score of 19 or more). Men were more likely to have cardiovascular disease. There were no other sex differences for medical conditions.

#### Ethnicity (Table 4)

Ethnicity was not associated with depression on any of the screening measures or ICD-10 depression diagnoses. The South Asian group was most likely to have type 2 diabetes, followed by the White British group. The Black group was the least likely to have type 2 diabetes. Hypertension was most common in the Black group. The White British group were most likely to have had or have cancer at the time of the interview. Autoimmune disorders were least common among the White British group. There were no other differences by specific condition or by levels of multi-morbidity.

### Univariate associations with ICD-10 diagnosis of moderate or severe depression

Block 1: Of all the demographic factors entered in stepwise backward logistic regression models (at *p* < 0.1 level), education and sex were retained. Compared with the reference group of those completing school, those having attended university or apprenticeships had a lower risk of depression (odds ratio 0.28, 95% CI: 0.08–0.98, *p* = 0.05), as did those who did not complete schooling (odds ratio 0.27, 95% CI: 0.08–0.94, *p* = 0.04). There was a trend for women to be more likely to have depression: odds ratio 2.14, 95% CI: 0.92–5.00, *p* = 0.07)

Block 2: Discrimination did not associate with depression. Three life events were associated with depression: loss of parent, spouse or child: odds ratio 5.05, 95% CI: 1.94–13.13, *p* < 0.01), marital separation (odds ratio 10.03, 95% CI: 1.89–53.08, *p* < 0.01) and facing major financial difficulties (odds ratio 3.66, 95% CI: 1.42–9.46, *p* < 0.01).

Block 3: Of long-term conditions, three were retained: obesity (odds ratio 3.89, 95% CI: 1.17–12.97, *p* = 0.07), COPD (odds ratio 5.25, 95% CI: 0.94–29.26, *p* = 0.06) and type 2 diabetes (odds ratio 2.53, 95% CI: 0.93–6.85, *p* = 0.07).

### Multivariate logistic regression showing associations with moderate or severe depression

The variables identified in the three blocks of backward stepwise regression models were entered into a multiple logistic regression model. We included age and sex to address the risk of residual confounding. The full regression model included educational level, traumatic life events (loss of parent, child, or spouse; major financial difficulties, marital difficulties) and chronic conditions (type 2 diabetes, obesity and COPD). Table 5 shows the full model, highlighting those reaching *p* ≤ 0.05: higher odds of depression was found among those with type 2 diabetes, those facing major financial crises and those who had experienced the loss of a parent, child or spouse. Lower odds of depression were found among those with university versus school-level education.

**Table 5:** Final logistic regression models: associations with moderate or severe depression

		Full model		
Demographic	*n*	Odds ratio	95% CI	*p*-value
Age				
<40	33	1		
40–60	106	0.74	0.11–5.26	0.77
>60	161	0.21	0.02–1.90	0.17
Gender				
Male	177	1		
Female	123	2.14	0.71–6.51	0.18
Education^a^				
School	133	1		
Degree/apprenticeship	77	0.18	0.03–1.02	0.05
None	76	0.46	0.11–1.92	0.29
Chronic obstructive pulmonary disease				
No	291	1		
Yes	8	4.7	0.4–55.07	0.22
Obesity				
No	277	1		
Yes	22	2.51	0.55–11.39	0.24
Diabetes II				
No	170	1		
Yes	133	5.32	1.34–20.76	0.02
Major financial crisis				
No	249	1		
Yes	51	3.51	1.04–11.87	0.04
Marital separation				
No	293	1		
Yes	7	5.34	0.70–40.90	0.11
Parent, child, spouse dies				
No	258	1		
Yes	42	3.62	1.09–12.0	0.04

*N* = 271, *X* ^2^ (d.f. = 10) = 41.20, *R* ^2^ = 0.0.29, *p* < 0.0001.

a. School set as reference as it is the largest group.

## Discussion

Depression is considered to be common in ESKD, a poor prognostic indicator, yet assessment and treatment is inconsistent.^7^ There is emerging evidence of benefits from antidepressants, psychological therapies and psychosocial interventions in patients living with CKD.^12,39^ Despite this, sometimes the treatment of depression in ESKD is contested on grounds of limited effectiveness and concerns about adverse effects in a frail patient population.^11,12^

Our main finding is that an ICD-10 diagnosis of moderate or severe depression, which warrants assertive treatment, is not as common as is usually proposed in the ESKD population. For example, we found a prevalence of 8%, whereas a careful meta-analysis reported a prevalence of 22.8% using structured diagnostic criteria.^40^ Our estimated prevalence of 8% is only twice that found in studies of the general population.^41^

Furthermore, screening and symptom measures which are used in clinical services (such as the HAMD or PHQ2 scores) grossly overestimate the prevalence of depression, so should always be followed by either a clinical assessment or a structured instrument.^40^ We highlight the importance of following up patients screening positive for depression with more accurate diagnostic assessments. The targeting of depression interventions to moderate or severe depression assessed by clinicians may help improve the risk–benefit ratio for these therapies.

Our findings suggest that financial strain and loss events are additional and important factors associated with depression. Therefore, holistic interventions (including, for example, practical financial advice and psychological support) are important aspects of care and future interventions including support for these will require further evaluation. Type 2 diabetes was also an important correlate of moderate to severe depression. Consequently, future studies could consider tailored interventions and care pathways for diabetic patients. Although there are concerns about the validity of structured and screening instruments in ethnically diverse populations,^23,24,26^ we did not find ethnic differences in the levels of depression. These findings encourage an individual and person-centred approach, taking account of factors relevant to each person’s biography and lived experiences of adversity and developing poor health.

### Limitations and strengths

The study was undertaken during the early phases of the COVID-19 pandemic, and this restricted access to the most vulnerable patients, potentially skewing results. Our measure of depression was based on the CIS-R, which is a well-established structured tool, which may have underestimated the true prevalence of depression in the highly diverse, deprived and urban population included in this study, and perhaps warrants more development in renal populations, although it has been previously tested in ethnically diverse samples.^26^ Against this, the CIS-R was being applied by an experienced clinical psychologist who could make judgements about the symptom endorsement, and who applied the algorithm in accord with guidance from the EMPIRIC study of ethnically diverse population samples which has been validated in similar contexts.^36,42^ Our algorithm excluded questions about appetite, weight loss and impairment, given that these, we found, were universally endorsed and difficult sometimes to discern diagnostic significance. This might have led to underestimates of diagnosed moderate or severe depression. We considered whether our careful exclusion of diagnostically confounding symptoms (e.g. appetite, weight and social impairment) might lead to underestimation of diagnosis. In a *post hoc* sensitivity analysis, we re-ran the algorithm as if each of these were considered present. For combined moderate and severe depression, we found 54 cases instead of 24, suggesting a prevalence of 18%, which is still much less than previous prevalence studies, even using structured instruments. It is critical to consider whether depression treatments should be targeted only following a structured instrument rather than screening instruments, and after excluding appetite, weight and social impairment symptoms. These confounding symptoms may explain the over-estimation of prevalence in previous studies and the reluctance of clinicians to treat depression in dialysis.

In our final models, women, the presence of obesity, and marital separation showed higher odds ratios but did not reach conventional levels of significance. However, these may still be important when considering personalised care and future research. We acknowledge the wider confidence intervals and smaller numbers of people with depression overall, and this may have reduced the power to detect importance differences; larger clinical samples and secondary analysis of population data may offer replication and more precise estimates. The original study design was to recruit up to 600 patients based on estimates of 350 existing patients and 250 new patients each year, whereby the recruitment period was to run over a year. However, the study was undertaken just as the COVID-19 pandemic led to a shutdown of all research studies, especially for vulnerable groups. Hence, we recruited as many patients as possible within the study period, and even after the pandemic, access was restricted for some time for vulnerable patients.

We did not assess pathways into care, and whether patients had received health promotional material or support prior to dialysis. This might be a useful approach for future research to identify preventive opportunities for depression and CKD.

### Implications

We report much lower prevalence of moderate or severe depression than has previously been reported in patients with ESKD receiving maintenance haemodialysis. We highlight the importance of following up patients with positive screening tools with more accurate diagnostic assessments, excluding appetite, weight and impairment questions. Future work should develop and test complex multi-component interventions to support ESKD patients facing financial difficulties, multiple losses events and bereavements, and those with other chronic conditions, especially type 2 diabetes.

## Supporting information

10.1192/bjo.2026.11039.sm001 Bhui et al. supplementary material 1 Bhui et al. supplementary material

10.1192/bjo.2026.11039.sm002 Bhui et al. supplementary material 2 Bhui et al. supplementary material

## Data Availability

The data is still undergoing analysis, but we welcome collaborations and suggestions for secondary analysis. The original ethics approval was not for secondary analysis, so further approvals would be required for non-intended analyses. We welcome collaborations to replicate and improve the methods, and the data are available for all reasonable requests.

## Supplementary material

The supplementary material is available online at https://doi.org/10.1192/bjo.2026.11039
