# Supplementary material for: Depression in dialysis patients with end-stage kidney disease: investigating the role of psychosocial stressors, co-occurring medical conditions and demographic influences
Source: BJPsych Open. 2026 May 11;12(3):e133. doi: 10.1192/bjo.2026.11039 (PMC13169048; doi:10.1192/bjo.2026.11039)
Supplement: Bhui et al. supplementary material 1 — Bhui et al. supplementary material [file S2056472426110394sup001.docx]

| \| **Annex A: Recruitment by centres for 294 of the 300 patients participating** (from log-books) \| \| \| \| \| \| \| --- \| --- \| --- \| --- \| --- \| --- \| \|  \|  \|  \|  \|  \|  \| \| **Unit** \| **Accepted** \| **Refused** \| **Excluded** \| **% of eligible recruited** \| \| \| Newham \| 101 \| 21 \| 9 \| 82 \|  \| \| Queens \| 56 \| 12 \| 3 \| 82 \|  \| \| King George \| 79 \| 14 \| 5 \| 85 \|  \| \| Whitecross (main) \| 43 \| 12 \| 3 \| 78 \|  \| \| Whitecross (Patience) \| 15 \| 3 \| 2 \| 83 \|  \| \| **Total** \| **294** \| **62** \| **22** \| **0.8258427** \|  \|   **Annex C: Depression by Sex, with and without somatic symptoms** | | | | | | | | |
| --- | --- | --- | --- | --- | --- | --- | --- | --- | --- | --- | --- | --- | --- | --- | --- | --- | --- | --- | --- | --- | --- | --- | --- | --- | --- | --- | --- | --- | --- | --- | --- | --- | --- | --- | --- | --- | --- | --- | --- | --- | --- | --- | --- | --- | --- | --- | --- | --- | --- | --- | --- | --- | --- | --- | --- | --- | --- | --- | --- | --- | --- | --- |
|  |  | **Total** |  | **Male** | **Female** | **X^2^** | **df** | **p** |
| Dep Mild No Somatic Sy | No | 276 (92) |  | 164 (92.7) | 112 (91.1) | 0.25 | 1 | 0.62 |
| (F3200) | Yes | 24 (8) |  | 13 (7.3) | 11 (8.9) |  |  |  |
| Dep Mild + Somatic Sy | No | 271 (90.3) |  | 165 (93.2) | 106 (86.2) | 4.12 | 1 | 0.04 |
| (F3201) | Yes | 29 (9.7) |  | 12 (6.8) | 17 (13.82) |  |  |  |
| Dep Mod or Severe: No Somatic Sy | No | 291 (97) |  | 172 (97.2) | 119 (96.8) | 0.05 | 1 | 0.83 |
| (F32.10) | Yes | 9 (3) |  | 5 (2.8) | 4 (3.3) |  |  |  |
| Dep Moderate or Severe: with Somatic Sy | No | 285 (95) |  | 172 (97.2) | 113 (91.9) | 4.3 | 1 | 0.04 |
| (F32.11 + F322) | Yes | 15 (5) |  | 5 (2.8) | 10 (8.1) |  |  |  |
