# Supplementary material for: Depression in dialysis patients with end-stage kidney disease: investigating the role of psychosocial stressors, co-occurring medical conditions and demographic influences
Source: BJPsych Open. 2026 May 11;12(3):e133. doi: 10.1192/bjo.2026.11039 (PMC13169048; doi:10.1192/bjo.2026.11039)
Supplement: Bhui et al. supplementary material 2 — Bhui et al. supplementary material [file S2056472426110394sup002.docx]

**Table of Algorithm Content for CIS-R evaluation of Depression ICD-10 Diagnostics.**

| **F32.00 Mild Depressive Episode without somatic symptoms**   1. Symptom duration ≥ 2 weeks DepJ > = 2 weeks   2. Two or more from:  • Depressed mood DepC = 1 and DepE>4 and DepF = 1 and DepI = 2  • Loss of interest DepD = 1  • Fatigue Fatsum ≥2  3. Two or three from:  • Reduced concentration Forgsum ≥2  • Reduced self-esteem IdeasG = 1  • Ideas of guilt IdeasF = 1  • Pessimism about future IdeasH = 1  • Suicidal ideas or acts SelfHmE = 1 or SelfHmG = 1  • Disturbed sleep Slpsum ≥2  • Diminished appetite🡪 Not evaluated as the overall CIS-R has not been done on the recommendations of the senior research team to not exhaust the patients.  4. Social impairment OverallA = 1🡪 Not requested during interview  5. Fewer than four from:  • Lack of normal pleasure/ interest DepD = 1  • Loss of normal emotional reactivity DepI = 1  • A.M. waking ≥ 2 hours early SleepH = 1  • Loss of libido IdeasB = 2  • Diurnal variation in mood IdeasA = 1 or 2  • Psychomotor agitation IdeasC=1  • Psychomotor retardation IdeasD = 1  • Diminished appetite 🡪 Not evaluated as the overall CIS-R has not been done on the recommendations of the senior research team to not exhaust the patients.  • Loss of =>5% body weight 🡪 Not evaluated as the overall CIS-R has not been done on the recommendations of the senior research team to not exhaust the patients.  6. Overall CIS-R score is significant CISband2 = 2🡪 Not evaluated as the overall CIS-R has not been done on the recommendations of the senior research team to not exhaust the patients. |
| --- |
| **F32.01 Mild Depressive Episode with somatic symptoms**   1. Symptom duration ≥ 2 weeks DepJ > = 2 weeks   2. Two or more from:  • Depressed mood DepC = 1 and DepE>4 and DepF = 1 and DepI = 2  • Loss of interest DepD = 1  • Fatigue Fatsum ≥2  3. Two or three from:  • Reduced concentration Forgsum ≥2  • Reduced self-esteem IdeasG = 1  • Ideas of guilt IdeasF = 1  • Pessimism about future IdeasH = 1  • Suicidal ideas or acts SelfHmE = 1 or SelfHmG = 1  • Disturbed sleep Slpsum ≥2  • Diminished appetite🡪 Not evaluated as the overall CIS-R has not been done on the recommendations of the senior research team to not exhaust the patients.  4. Social impairment OverallA = 1🡪 Not requested during interview  5. Four or more from:  • Lack of normal pleasure/ interest DepD=1  • Loss of normal emotional reactivity DepI = 1  • A.M. waking ≥ 2 hours early SleepH = 1  • Loss of libido IdeasB = 2  • Diurnal variation in mood IdeasA = 1 or 2  • Psychomotor agitation IdeasC = 1  • Psychomotor retardation IdeasD = 1  • Diminished appetite 🡪 Not evaluated as the overall CIS-R has not been done on the recommendations of the senior research team to not exhaust the patients.  • Loss of =>5% body weight 🡪 Not evaluated as the overall CIS-R has not been done on the recommendations of the senior research team to not exhaust the patients.  6. Overall CIS-R score is significant CISband2 = 2🡪 Not evaluated as the overall CIS-R has not been done on the recommendations of the senior research team to not exhaust the patients. |
| **F32.10 Moderate Depressive Episode without somatic symptoms**   1. Symptom duration ≥ 2 weeks DepJ > = 2 weeks   2. Two or more from:  • Depressed mood DepC = 1 and DepE>4 and DepF = 1 and DepI = 2  • Loss of interest DepD = 1  • Fatigue Fatsum ≥2  3. Four or more from:  • Reduced concentration Forgsum ≥2  • Reduced self-esteem IdeasG = 1  • Ideas of guilt IdeasF = 1  • Pessimism about future IdeasH = 1  • Suicidal ideas or acts SelfHmE = 1 or SelfHmG = 1  • Disturbed sleep Slpsum ≥2  • Diminished appetite🡪 Not evaluated as the overall CIS-R has not been done on the recommendations of the senior research team to not exhaust the patients.  4. Social impairment OverallA = 1🡪 Not requested during interview  5. Fewer than four from:  • Lack of normal pleasure/ interest DepD = 1  • Loss of normal emotional reactivity DepI = 1  • A.M. waking ≥ 2 hours early SleepH = 1  • Loss of libido IdeasB = 2  • Diurnal variation in mood IdeasA = 1 or 2  • Psychomotor agitation IdeasC=1  • Psychomotor retardation IdeasD = 1  • Diminished appetite 🡪 Not evaluated as the overall CIS-R has not been done on the recommendations of the senior research team to not exhaust the patients.  • Loss of =>5% body weight 🡪 Not evaluated as the overall CIS-R has not been done on the recommendations of the senior research team to not exhaust the patients.  6. Overall CIS-R score is significant CISband2 = 2🡪 Not evaluated as the overall CIS-R has not been done on the recommendations of the senior research team to not exhaust the patients. |
| **F32.11 Moderate Depressive Episode with somatic symptoms**   1. Symptom duration ≥ 2 weeks DepJ > = 2 weeksf 2. Two or more from:   • Depressed mood DepC = 1 and DepE>4 and DepF = 1 and DepI = 2  • Loss of interest DepD = 1  • Fatigue Fatsum ≥2  3. Four or more from:  • Reduced concentration Forgsum ≥2  • Reduced self-esteem IdeasG = 1  • Ideas of guilt IdeasF = 1  • Pessimism about future IdeasH = 1  • Suicidal ideas or acts SelfHmE = 1 or SelfHmG = 1  • Disturbed sleep Slpsum ≥2  • Diminished appetite 🡪 Not evaluated as the overall CIS-R has not been done on the recommendations of the senior research team to not exhaust the patients.   1. Social impairment OverallA = 1 🡪 Not requested during interview   5. Fewer than four from: < 4  • Lack of normal pleasure/ interest DepD = 1  • Loss of normal emotional reactivity DepI = 1  • A.M. waking ≥ 2 hours early SleepH = 1  • Loss of libido IdeasB = 2  • Diurnal variation in mood IdeasA = 1 or 2  • Psychomotor agitation IdeasC=1  • Psychomotor retardation IdeasD = 1  • Diminished appetite 🡪 Not evaluated as the overall CIS-R has not been done on the recommendations of the senior research team to not exhaust the patients.  • Loss of =>5% body weight 🡪 Not evaluated as the overall CIS-R has not been done on the recommendations of the senior research team to not exhaust the patients.  6. Overall CIS-R score is significant CISband2 = 2 🡪 Not evaluated as the overall CIS-R has not been done on the recommendations of the senior research team to not exhaust the patients. |
| **F32.2 Severe Depressive Disorder**   1. Symptom duration ≥ 2 weeks DepJ > = 2 weeks   2. Two or more from:  • Depressed mood DepC = 1 and DepE>4 and DepF = 1 and DepI = 2  • Loss of interest DepD = 1  • Fatigue Fatsum ≥2  3. Four or more from:  • Reduced concentration Forgsum ≥2  • Reduced self-esteem IdeasG = 1  • Ideas of guilt IdeasF = 1  • Pessimism about future IdeasH = 1  • Suicidal ideas or acts SelfHmE = 1 or SelfHmG = 1  • Disturbed sleep Slpsum ≥2  • Diminished appetite-🡪 Not evaluated as the overall CIS-R has not been done on the recommendations of the senior research team to not exhaust the patients.  4. Social impairment OverallA = 1 🡪 Not requested during Interview  5. Four or more from:  • Lack of normal pleasure/ interest DepD = 1  • Loss of normal emotional reactivity DepI = 1  • A.M. waking ≥ 2 hours early SleepH = 1  • Loss of libido IdeasB = 2  • Diurnal variation in mood IdeasA = 1 or 2  • Psychomotor agitation IdeasC=1  • Psychomotor retardation IdeasD = 1  • Diminished appetite 🡪 Not evaluated as the overall CIS-R has not been done on the recommendations of the senior research team to not exhaust the patients.  • Loss of =>5% body weight 🡪 Not evaluated as the overall CIS-R has not been done on the recommendations of the senior research team to not exhaust the patients.  6. Overall CIS-R score is significant CISband2 = 2 🡪 Not evaluated as the overall CIS-R has not been done on the recommendations of the senior research team to not exhaust the patients. |

**Table of Correspondence Code for the CIS-R algorithm for Depression**

| **CIS-R section** | **CIS-R question** | **Biomedical survey variable name** | **Scoring for Symptom scores** |
| --- | --- | --- | --- |
| Appetite Section | Not explored | | |
| Somatic Section |  | | |
|  | "In the past seven days, including last (Day of week), on how many days have you noticed the ache or pain/discomfort?" | SomaD | 4 days or more = 1 |
|  | "In total did the ache or pain/discomfort last for more than 3 hours on any day in the past week/on that day?" | SomaE | More than 3 hours on any day = 1 |
|  | "In the past week, has the ache or pain/discomfort been" | SomaF | Symptoms very unpleasants = 1 |
|  | "Has the ache or pain/discomfort bothered you when you were doing something interesting in the past week?" | SomaG | Bothered when doing something interesting = 1 |
| Fatigue Section |  |  |  |
|  | "In the past seven days incuding last (days of the week) on how many days have you felt tired/lacking in energy?" | FatigE | 4 days or more = 1 |
|  | "Have you felt tired/lacking in energy for more than 3 hours in total on any day in the past week?" | FatigF | Yes = 1 |
|  | "Have you felt so tired/lacking in energy that you've had to push yourself to fet things done during the past week?" | FatigG | Yes on at least one occasion = 1 |
|  | "Have you felt tired/lacking in energy when doing things that you enjoy during the past week?" | FatigH | Yes at least once = 1 **OR**  Yes = 1  Nb: *Either fatigH asked or fatigI asked – not both* 1 |
|  | "Have you in the past week felt tired/lacking in energy when doing things that you used to enjoy?” | FatigI |  |
|  |  | Fatsum = FatigE+FatigF+FatigG+FatigH (max score =4) | |
| Concentration / Forgetfulness |  | | |
|  | "Since last (DAY OF WEEK), on how many days have you noticed problems with your concentration/memory?" | ForgetC | 4 days or more = 1 |
|  | "In the past week could you concentrate on a TV programme, read a newspaper article or talk to someone without your mind wandering?" | ForgetD | No, not always = 2 |
|  | "In the past week, have these problems with your concentration actuallty stopped you from getting on with things you used to do or would like to do?" | ForgetE | Yes = 1 |
|  | "(Earlier you said you have been forgetting things.) Have you forgotten anything important in the past seven days?" | ForgetF | Yes = 1 |
|  |  | Forgsum= ForgC+ForgD (on 1)+ForgE+ForgF (max score =4) | |
| Sleep Problems |  | | |
|  | "On how many of the past seven nights did you have problems with your sleep?" | SleepC | 4 nights or more = 1 |
|  | "How long did you spend trying to get to sleep…" | sleepF | At least ¼ hr < than 1 hour =2  At least 1hr but < than 3hrs =3  3 hrs or more = 4 |
|  | "In the past week, on how many nights did you spend 3 or more hours trying to get to sleep" | SleepG | 4 nights or more = 1 |
|  | "Thinking about the night you slept the longest in the past week, how much longer did you sleep compared with how long you normally sleep for?" | sleepI | At least ¼ hr < than 1 hour =1  At least 1hr but < than 3hrs =2  3 hrs or more = 2 |
|  | "In the past week, on how many nights did you sleep for more than 3 hours longer than you usually do?" | SleepJ | 4 nights or more =1 |
|  |  | SleepSum = SleepC+SleepF+SleepG+SleepI+SleepJ (max score =5) | |
| Irritability |  | | |
|  | "Since last (day of week) on how many days have you felt irritable or short tempered/angry?" | IrritC | 4 days or more = 1 |
|  | "In total, have you felt irritable or short tempered/angry for more than one hour (on any day in the past week?)" | IrritE | Yes = 1 |
|  | During the past week, have you felt so irritable or short tempered/angry that you have wanted to shout at someone even if you haven't actually shouted?" | IrritF | Yes = 1 |
|  | "Do you think it was justified?" | IrritI | No, not justified = 2 **OR**  No, at least once was unjustified = 2 |
|  | "Do you think this was justified on every occasion?" | IrritJ |  |
| Depressed Mood |  | | |
|  | "In the past week, have you been able to enjoy or take an interest in things as much as usual?" | DepD | No = 1 |
|  | "Since last (Day of Week), on how many days have you felt sad, miserable or depressed/unable to enjoy or take an interest in things?" | DepE | 4 days or more = 1 |
|  | "Have you felt sad, miserable or depressed/unable to enjoy or take an interest in things for more than 3 hours in total (on any day of the last week)?" | DepF | Yes = 1 |
|  | "In the past week, when you felt sad, miserable or depressed/unable to enjoy or take an interest in things, did you ever become happier when something nice happened, or when you were in company?" | DepI | No = 1 |
| Depressive Ideas |  | | |
|  | "Now, thinking about the past seven days have you on at least one occasion felt guilty or blamed yourself when things went wrong when it hasn't been your fault?" | IdeasF | Yes = 1 |
|  | "During the past week, have you been feeling you are not as good as other people?" | IdeasG | Yes = 1 |
|  | "Have you felt hopeless at all during the past seven days, for instance about your future?" | IdeasH | Yes = 1 |
|  | "In the past week have you felt that life isn't worth living?" | SelfHmB1 | Yes=1 |
|  | "In the past week, have you thought of killing yourself?" | SelfHmD2 | Yes=1 |

**Summary of “R” Codes used to generate CIS-R ICD diagnostics.**

setwd("C:/Users/christophe.clesse/Downloads/CISR")

list.files()

data = read.csv("INTREPID_barts_data_cleaned_final_csv.csv", header = TRUE, sep = ",", as.is=T)

data <-fread("INTREPID_barts_data_cleaned_final_csv.csv")

write.table(data, file = "INTREPID_barts_data_cleaned_final.txt", sep = "\t")

library(dplyr)

library(plyr)

#### Creating sum columns by Adding columns values while ignoring NAs###

###Columns of interest have been matched with the CIS-R and also with the codebook###

#####Creation of DepJ####

##G10 is DepJ##

count(data$CISR_G10_Depression_How_long_depressed_anhedonic)

data$DepJ <- mapvalues(data$CISR_G10_Depression_How_long_depressed_anhedonic, from=c(2,3,4,5,NA), to=c(1,1,1,1,NA))

count(data$DepJ)

#DepJ is coded 1 if depressed 2 weeks or more and NA if not

#####Creation of DepC###

#G4 is DepC

count(data$CISR_G4_Depression_Last_week_depressed_miserable_sad)

count(data$DepC)

data$DepC <- mapvalues(data$CISR_G4_Depression_Last_week_depressed_miserable_sad, from=c(1,2,NA), to=c(1,0,NA))

count(data$DepC)

#If depressed last week, coded 1 otherwise coded 0.

### Creation of DepD###

#G5 is DepD#

count(data$CISR_G5_Depression_Last_week_Able_to_enjoy_take_interest)

data$DepD <- mapvalues(data$CISR_G5_Depression_Last_week_Able_to_enjoy_take_interest, from=c(1,2,NA), to=c(1,0,NA))

count(data$DepD)

##### Creation of DepE #####

#G6 is DepE#

count(data$CISR_G6_Depression_How_many_days_felt_depressed_anhedonic)

data$DepE <- mapvalues(data$CISR_G6_Depression_How_many_days_felt_depressed_anhedonic, from=c(1,2,3,NA), to=c(1,0,0,NA))

count(data$DepE)

#If felt depressed more than 4 days, coded 1#

##### Creation of DepF #####

count(data$CISR_G7_Depression_Depressed_anhedonic_more_than_3_hours)

data$DepF <- mapvalues(data$CISR_G7_Depression_Depressed_anhedonic_more_than_3_hours, from=c(1,2,NA), to=c(1,0,NA))

count(data$DepF)

#If depressed more than 3 hours, coded 1#

### Creation of DepI

# G9 is DepI #

count(data$CISR_G9_Depression_Become_happier_when_nice_thing_happened)

data$DepI <- mapvalues(data$CISR_G9_Depression_Become_happier_when_nice_thing_happened, from=c(1,2,NA), to=c(1,2,NA))

count(data$DepI)

#If couldn't become happier, coded 2 , If could happier coded 1 #

### Creation of IdeasG

# H5 is IdeasG #

count(data$CISR_H5_Depressive_ideas_2t_as_good_as_other_people)

data$IdeasG <- mapvalues(data$CISR_H5_Depressive_ideas_2t_as_good_as_other_people, from=c(1,2,NA), to=c(1,0,NA))

count(data$IdeasG)

#If feels not as good as other, coded 1#

### Creation of IdeasF

# H4 is IdeasF #

count(data$CISR_H4_Depressive_ideas_Guilty_blame_yourself)

data$IdeasF <- mapvalues(data$CISR_H4_Depressive_ideas_Guilty_blame_yourself, from=c(1,2,NA), to=c(1,0,NA))

count(data$IdeasF)

#If feels guilty, coded 1#

### Creation of IdeasH###

# H6 is IdeasH #

count(data$CISR_H6_Depressive_ideas_Hopeless_last_seven_days)

data$IdeasH <- mapvalues(data$CISR_H6_Depressive_ideas_Hopeless_last_seven_days, from=c(1,2,NA), to=c(1,0,NA))

count(data$IdeasH)

#If feels hopeless last 7 days, coded 1#

### Creation of SelfHmE###

# H8 is SelfHmE #

count(data$CISR_H8_Depressive_ideas_Life_isnt_worth_living)

data$SelfHmE <- mapvalues(data$CISR_H8_Depressive_ideas_Life_isnt_worth_living, from=c(1,2,3,NA), to=c(1,0,0,NA))

count(data$SelfHmE)

#If feels hopeless last 7 days, coded 1#

### Creation of SelfHmG###

# H9 is SelfHmG #

count(data$CISR_H9_Depressive_ideas_thought_killing_yourself)

data$SelfHmG <- mapvalues(data$CISR_H9_Depressive_ideas_thought_killing_yourself, from=c(1,2,NA), to=c(1,0,NA))

count(data$SelfHmG)

#If thought about kill itself, coded 1#

### Creation of SleepH###

# D7 is SleepH #

count(data$CISR_D7_Sleep_Wake_up_and_cant_sleep_again)

data$SleepH <- mapvalues(data$CISR_D7_Sleep_Wake_up_and_cant_sleep_again, from=c(1,2,NA), to=c(1,0,NA))

count(data$SleepH)

#If wake up 2 hours early and can't go back to sleep, coded 1#

### Creation of IdeasB###

# H2 is IdeasB #

count(data$CISR_H2_Depressive_ideas_Interrest_in_sex)

data$IdeasB <- mapvalues(data$CISR_H2_Depressive_ideas_Interrest_in_sex, from=c(1,2,3,4,NA), to=c(0,1,0,0,NA))

count(data$IdeasB)

#If coded 1, decreased in sex#

### Creation of IdeasA###

# H1 is IdeasA #

count(data$CISR_H1_Depressive_ideas_Morning_evening_no_difference)

data$IdeasA <- mapvalues(data$CISR_H1_Depressive_ideas_Morning_evening_no_difference, from=c(1,2,3,NA), to=c(1,1,0,NA))

count(data$IdeasA)

#If coded 1, diurnal change of mood#

### Creation of IdeasC###

# H3a is IdeasC #

count(data$CISR_H3a_Depressive_ideas_Restless_couldnt_sit_still)

data$IdeasC <- mapvalues(data$CISR_H3a_Depressive_ideas_Restless_couldnt_sit_still, from=c(1,2,NA), to=c(1,0,NA))

count(data$IdeasC)

#If coded 1, restless or psychomotor agitation#

### Creation of IdeasD###

# H3a is IdeasD #

count(data$CISR_H3b_Depressive_ideas_Doing_things_slowly)

data$IdeasD <- mapvalues(data$CISR_H3b_Depressive_ideas_Doing_things_slowly, from=c(1,2,NA), to=c(1,0,NA))

count(data$IdeasD)

#If coded 1, restless or psychomotor agitation#

##Fatsum##

#(NB due to a misreading, here FatigI is named FatigL without capital letter for the L)#

count(data$Fatigl)

data$FatigEs <- mapvalues(data$FatigE, from=c(1,NA), to=c(1,NA))

data$FatigFs <- mapvalues(data$FatigF, from=c(1,NA), to=c(1,NA))

data$FatigGs <- mapvalues(data$FatigG, from=c(1,NA), to=c(1,NA))

data <-data %>% mutate(FatigHLs = rowSums(select(., FatigH, Fatigl), na.rm = TRUE))

count(data$FatigGs)

data <-data %>% mutate(Fatsum = rowSums(select(., FatigEs, FatigFs, FatigGs,FatigHLs), na.rm = TRUE))

count(data$Fatsum)

##Slpsum##

count(data$SleepJ)

data$SleepCs <- mapvalues(data$SleepC, from=c(1,NA), to=c(1,NA))

data$SleepFs <- mapvalues(data$SleepF, from=c(2,3,4,NA), to=c(1,1,1,NA))

data$SleepGs <- mapvalues(data$SleepG, from=c(1,NA), to=c(1,NA))

data$Sleepls <- mapvalues(data$Sleepl, from=c(2,3,4,NA), to=c(1,1,1,NA))

data$SleepJs <- mapvalues(data$SleepJ, from=c(1,NA), to=c(1,NA))

data <-data %>% mutate(Slpsum = rowSums(select(., SleepCs, SleepFs,SleepGs, Sleepls, SleepJs), na.rm = TRUE))

count(data$Slpsum)

##Forgsum##

data$ForgetCs <- mapvalues(data$ForgetC, from=c(1,NA), to=c(1,NA))

data$ForgetDs <- mapvalues(data$ForgetD, from=c(2,NA), to=c(1,NA))

data$ForgetEs <- mapvalues(data$ForgetE, from=c(1,NA), to=c(1,NA))

data$ForgetFs <- mapvalues(data$ForgetF, from=c(1,NA), to=c(1,NA))

data <-data %>% mutate(Forgsum = rowSums(select(., ForgetCs, ForgetDs,ForgetEs, ForgetFs), na.rm = TRUE))

count(data$Forgsum)

##################################################

############### CIS-R Formula ######################

##################################################

library(tidyverse)

setwd("C:/Users/christophe.clesse/Downloads/CISR")

library(dplyr)

library(plyr)

####Removing the NA and changing it to 0 as no impact on control group####

data$DepJ <- mapvalues(data$DepJ, from=c(NA), to=c(0))

data$DepC <- mapvalues(data$DepC, from=c(NA), to=c(0))

data$DepE <- mapvalues(data$DepE, from=c(NA), to=c(0))

data$DepF <- mapvalues(data$DepF, from=c(NA), to=c(0))

data$DepI <- mapvalues(data$DepI, from=c(NA), to=c(0))

data$IdeasG <- mapvalues(data$IdeasG, from=c(NA), to=c(0))

data$IdeasF <- mapvalues(data$IdeasF, from=c(NA), to=c(0))

data$IdeasH <- mapvalues(data$IdeasH, from=c(NA), to=c(0))

data$SelfHmE <- mapvalues(data$SelfHmE, from=c(NA), to=c(0))

data$SelfHmG <- mapvalues(data$SelfHmG, from=c(NA), to=c(0))

data$SleepH <- mapvalues(data$SleepH, from=c(NA), to=c(0))

data$DepD <- mapvalues(data$DepD, from=c(NA), to=c(0))

data$IdeasB <- mapvalues(data$IdeasB, from=c(NA), to=c(0))

data$IdeasA <- mapvalues(data$IdeasA, from=c(NA), to=c(0))

data$IdeasC <- mapvalues(data$IdeasC, from=c(NA), to=c(0))

data$IdeasD <- mapvalues(data$IdeasD, from=c(NA), to=c(0))

data$DepI <- mapvalues(data$DepI, from=c(1,2,NA), to=c(NA,1,NA))

count(data$DepI)

##################################################################

################# Creation of diagnostics #############################

##################################################################

library(tidyverse)

intrepid <- data

#####################################################################

###### F32.00 Mild Depressive Episode without somatic symptoms ##############

#####################################################################

#Conditon1

count(intrepid$DepJ)

intrepid$condition1 <- intrepid$DepJ == 1

count(intrepid$condition1)

#Condition 2: Two or more from:

# Depressed mood DepC = 1 and DepE>4 and DepF = 1 and DepI = 2

#(DepC, DepE, DepF are coded 1 in case conditions are matching)

# Loss of interest DepD = 1

# Fatigue Fatsum sup or equal to 2

intrepid$condition2 <- (intrepid$DepE * intrepid$DepF * intrepid$DepI + intrepid$DepD + intrepid$DepC + (intrepid$Fatsum >= 2)) >= 2

count(intrepid$condition2)

#Condition 3: Two or three from:

# Reduced concentration Forgsum ≥2

#Reduced self-esteem IdeasG = 1

#Ideas of guilt IdeasF = 1

#Pessimism about future IdeasH = 1

#Suicidal ideas or acts SelfHmE = 1 or SelfHmG = 1

#Disturbed sleep Slpsum ≥2

intrepid$condition3 <- ((intrepid$Forgsum>=2) + (intrepid$IdeasG == 1) + (intrepid$IdeasF == 1) + (intrepid$IdeasH == 1) + (intrepid$SelfHmE == 1) * (intrepid$SelfHmG == 1) + (intrepid$Slpsum >=2)) %in% c(2,3)

count(intrepid$condition3)

#Condition 4: Fewer than four from:

#Lack of normal pleasure/ interest DepD = 1

#Loss of normal emotional reactivity DepI = 1

#A.M. waking ≥ 2 hours early SleepH = 1

#Loss of libido IdeasB = 2 (It is coded 1 in case conditions are matching to make it easier)

#Diurnal variation in mood IdeasA = 1 or 2 (is coded 1 in case conditions are matching to make it easier)

#Psychomotor agitation IdeasC=1

#Psychomotor retardation IdeasD = 1

intrepid$condition4 <- ((intrepid$DepD == 1) + (intrepid$DepI == 1) + (intrepid$SleepH == 1) + (intrepid$IdeasB ==1) + (intrepid$IdeasA == 1) + (intrepid$IdeasC == 1) + (intrepid$IdeasD == 1)) < 4

count(intrepid$condition4)

##Final diagnosis##

intrepid$F3200Mild_Depressive_Episode_without_somatic_symptoms <- intrepid$condition1 * intrepid$condition2 * intrepid$condition3 * intrepid$condition4

count(intrepid$F3200Mild_Depressive_Episode_without_somatic_symptoms)

## 24 persons have a depression of F32.00##

#####################################################################

####### F32.01 Mild Depressive Episode with somatic symptoms ################

#####################################################################

#Conditon1

count(intrepid$DepJ)

intrepid$conditionF3201A <- intrepid$DepJ == 1

count(intrepid$conditionF3201A)

#Condition 2: Two or more from:

# Depressed mood DepC = 1 and DepE>4 and DepF = 1 and DepI = 2

#(DepC, DepE, DepF are coded 1 in case conditions are matching)

# Loss of interest DepD = 1

# Fatigue Fatsum sup or equal to 2

intrepid$conditionF3201B <- (intrepid$DepE * intrepid$DepF * intrepid$DepI + intrepid$DepD + intrepid$DepC + (intrepid$Fatsum >= 2)) >= 2

count(intrepid$conditionF3201B)

#Condition 3: Two or three from:

# Reduced concentration Forgsum ≥2

#Reduced self-esteem IdeasG = 1

#Ideas of guilt IdeasF = 1

#Pessimism about future IdeasH = 1

#Suicidal ideas or acts SelfHmE = 1 or SelfHmG = 1

#Disturbed sleep Slpsum ≥2

intrepid$conditionF3201C <- ((intrepid$Forgsum>=2) + (intrepid$IdeasG == 1) + (intrepid$IdeasF == 1) + (intrepid$IdeasH == 1) + (intrepid$SelfHmE == 1) * (intrepid$SelfHmG == 1) + (intrepid$Slpsum >=2)) %in% c(2,3)

count(intrepid$conditionF3201C)

#Condition 4: Four or more from:

#Lack of normal pleasure/ interest DepD = 1

#Loss of normal emotional reactivity DepI = 1

#A.M. waking ≥ 2 hours early SleepH = 1

#Loss of libido IdeasB = 2 (It is coded 1 in case conditions are matching to make it easier)

#Diurnal variation in mood IdeasA = 1 or 2 (is coded 1 in case conditions are matching to make it easier)

#Psychomotor agitation IdeasC=1

#Psychomotor retardation IdeasD = 1

intrepid$conditionF3201D <- ((intrepid$DepD == 1) + (intrepid$DepI == 1) + (intrepid$SleepH == 1) + (intrepid$IdeasB ==1) + (intrepid$IdeasA == 1) + (intrepid$IdeasC == 1) + (intrepid$IdeasD == 1)) >= 4

count(intrepid$conditionF3201D)

##Final diagnosis##

intrepid$F3201Mild_Depressive_Episode_with_somatic_symptoms <- intrepid$conditionF3201A * intrepid$conditionF3201B * intrepid$conditionF3201C * intrepid$conditionF3201D

count(intrepid$F3201Mild_Depressive_Episode_with_somatic_symptoms)

## 29 persons have a diagnostic of F32.01##

############################################################################

####### F32.10 Moderate Depressive Episode without somatic symptoms ################

############################################################################

#Conditon1

count(intrepid$DepJ)

intrepid$conditionF3210A <- intrepid$DepJ == 1

count(intrepid$conditionF3210A)

#Condition 2: Two or more from:

# Depressed mood DepC = 1 and DepE>4 and DepF = 1 and DepI = 2

#(DepC, DepE, DepF are coded 1 in case conditions are matching)

# Loss of interest DepD = 1

# Fatigue Fatsum sup or equal to 2

intrepid$conditionF3210B <- (intrepid$DepE * intrepid$DepF * intrepid$DepI + intrepid$DepD + intrepid$DepC + (intrepid$Fatsum >= 2)) >= 2

count(intrepid$conditionF3210B)

#Condition 3: Four or more from:

#Reduced concentration Forgsum ≥2

#Reduced self-esteem IdeasG = 1

#Ideas of guilt IdeasF = 1

#Pessimism about future IdeasH = 1

#Suicidal ideas or acts SelfHmE = 1 or SelfHmG = 1

#Disturbed sleep Slpsum ≥2

intrepid$conditionF3210C <- ((intrepid$Forgsum>=2) + (intrepid$IdeasG == 1) + (intrepid$IdeasF == 1) + (intrepid$IdeasH == 1) + (intrepid$SelfHmE == 1) * (intrepid$SelfHmG == 1) + (intrepid$Slpsum >=2)) >=4

count(intrepid$conditionF3210C)

#Condition 4: Fewer than 4 from:

#Lack of normal pleasure/ interest DepD = 1

#Loss of normal emotional reactivity DepI = 1

#A.M. waking ≥ 2 hours early SleepH = 1

#Loss of libido IdeasB = 2 (It is coded 1 in case conditions are matching to make it easier)

#Diurnal variation in mood IdeasA = 1 or 2 (is coded 1 in case conditions are matching to make it easier)

#Psychomotor agitation IdeasC=1

#Psychomotor retardation IdeasD = 1

intrepid$conditionF3210D <- ((intrepid$DepD == 1) + (intrepid$DepI == 1) + (intrepid$SleepH == 1) + (intrepid$IdeasB ==1) + (intrepid$IdeasA == 1) + (intrepid$IdeasC == 1) + (intrepid$IdeasD == 1)) < 4

count(intrepid$conditionF3210D)

##Final diagnosis##

intrepid$F3210Moderate_Depressive_Episode_without_somatic_symptoms <- intrepid$conditionF3210A * intrepid$conditionF3210B * intrepid$conditionF3210C * intrepid$conditionF3210D

count(intrepid$F3210Moderate_Depressive_Episode_without_somatic_symptoms)

### 9 individuals have moderate depression without somatic symptoms (F32.10)

############################################################################

######### F32.11 Moderate Depressive Episode with somatic symptoms #################

############################################################################

#Condition1

count(intrepid$DepJ)

intrepid$conditionF3211A <- intrepid$DepJ == 1

count(intrepid$conditionF3211A)

#Condition 2: Two or more from:

# Depressed mood DepC = 1 and DepE>4 and DepF = 1 and DepI = 2

#(DepC, DepE, DepF are coded 1 in case conditions are matching)

# Loss of interest DepD = 1

# Fatigue Fatsum sup or equal to 2

intrepid$conditionF3211B <- (intrepid$DepE * intrepid$DepF * intrepid$DepI + intrepid$DepD + intrepid$DepC + (intrepid$Fatsum >= 2)) >= 2

count(intrepid$conditionF3211B)

#Condition 3: Four or more from:

#Reduced concentration Forgsum ≥2

#Reduced self-esteem IdeasG = 1

#Ideas of guilt IdeasF = 1

#Pessimism about future IdeasH = 1

#Suicidal ideas or acts SelfHmE = 1 or SelfHmG = 1

#Disturbed sleep Slpsum ≥2

intrepid$conditionF3211C <- ((intrepid$Forgsum>=2) + (intrepid$IdeasG == 1) + (intrepid$IdeasF == 1) + (intrepid$IdeasH == 1) + (intrepid$SelfHmE == 1) * (intrepid$SelfHmG == 1) + (intrepid$Slpsum >=2)) >=4

count(intrepid$conditionF3211C)

#Condition 4: Four or more from:

#Lack of normal pleasure/ interest DepD = 1

#Loss of normal emotional reactivity DepI = 1

#A.M. waking ≥ 2 hours early SleepH = 1

#Loss of libido IdeasB = 2 (It is coded 1 in case conditions are matching to make it easier)

#Diurnal variation in mood IdeasA = 1 or 2 (is coded 1 in case conditions are matching to make it easier)

#Psychomotor agitation IdeasC=1

#Psychomotor retardation IdeasD = 1

intrepid$conditionF3211D <- ((intrepid$DepD == 1) + (intrepid$DepI == 1) + (intrepid$SleepH == 1) + (intrepid$IdeasB ==1) + (intrepid$IdeasA == 1) + (intrepid$IdeasC == 1) + (intrepid$IdeasD == 1)) >= 4

count(intrepid$conditionF3211D)

##Final diagnosis##

intrepid$F3211Moderate_Depressive_Episode_with_somatic_symptoms <- intrepid$conditionF3211A * intrepid$conditionF3211B * intrepid$conditionF3211C * intrepid$conditionF3211D

count(intrepid$F3211Moderate_Depressive_Episode_with_somatic_symptoms)

#### 15 individuals have a diagnosis of F32.11 ##

############################################################################

##################### F32.2 Severe Depressive Disorder ###########################

############################################################################

#Conditon1

count(intrepid$DepJ)

intrepid$conditionF322A <- intrepid$DepJ == 1

count(intrepid$conditionF322A)

#Condition 2: All three form:

# Depressed mood DepC = 1 and DepE>4 and DepF = 1 and DepI = 2

#(DepC, DepE, DepF are coded 1 in case conditions are matching)

# Loss of interest DepD = 1

# Fatigue Fatsum sup or equal to 2

intrepid$conditionF322B <- (intrepid$DepE * intrepid$DepF * intrepid$DepI + intrepid$DepD + intrepid$DepC + (intrepid$Fatsum >= 2)) ==3

count(intrepid$conditionF322B)

#Condition 3: Four or more from:

#Reduced concentration Forgsum ≥2

#Reduced self-esteem IdeasG = 1

#Ideas of guilt IdeasF = 1

#Pessimism about future IdeasH = 1

#Suicidal ideas or acts SelfHmE = 1 or SelfHmG = 1

#Disturbed sleep Slpsum ≥2

intrepid$conditionF322C <- ((intrepid$Forgsum>=2) + (intrepid$IdeasG == 1) + (intrepid$IdeasF == 1) + (intrepid$IdeasH == 1) + (intrepid$SelfHmE == 1) * (intrepid$SelfHmG == 1) + (intrepid$Slpsum >=2)) >=4

count(intrepid$conditionF322C)

#Condition 4: Four or more from:

#Lack of normal pleasure/ interest DepD = 1

#Loss of normal emotional reactivity DepI = 1

#A.M. waking ≥ 2 hours early SleepH = 1

#Loss of libido IdeasB = 2 (It is coded 1 in case conditions are matching to make it easier)

#Diurnal variation in mood IdeasA = 1 or 2 (is coded 1 in case conditions are matching to make it easier)

#Psychomotor agitation IdeasC=1

#Psychomotor retardation IdeasD = 1

intrepid$conditionF322D <- ((intrepid$DepD == 1) + (intrepid$DepI == 1) + (intrepid$SleepH == 1) + (intrepid$IdeasB ==1) + (intrepid$IdeasA == 1) + (intrepid$IdeasC == 1) + (intrepid$IdeasD == 1)) >=4

count(intrepid$conditionF322D)

##Final diagnosis##

intrepid$F322Severe_Depressive_Disorder <- intrepid$conditionF322A * intrepid$conditionF322B * intrepid$conditionF322C * intrepid$conditionF322D

count(intrepid$F322Severe_Depressive_Disorder)

#### 11 Individuals have a F32.2 diagnostic ####

data <-fread("INTREPID_barts_data_cleaned_final_csv.csv")

####################################################################################### ######### Exploration for Severity in diagnostics ########################################################################################################################################################

##### Mild Depressive Episode #####

intrepid$Mild_Depressive_Episode <- intrepid$F3200Mild_Depressive_Episode_without_somatic_symptoms | intrepid$F3201Mild_Depressive_Episode_with_somatic_symptoms

count(intrepid$Mild_Depressive_Episode)

##53 with mild depression##

####Moderate Depressive Episode #####

intrepid$Moderate_Depressive_Episode <- intrepid$F3211Moderate_Depressive_Episode_with_somatic_symptoms | intrepid$F3210Moderate_Depressive_Episode_without_somatic_symptoms

count(intrepid$Moderate_Depressive_Episode)

##24 with moderate depression##

##### Depressed CISR #####

intrepid$Depression_CISR <- intrepid$F3211Moderate_Depressive_Episode_with_somatic_symptoms | intrepid$F3210Moderate_Depressive_Episode_without_somatic_symptoms |intrepid$F3200Mild_Depressive_Episode_without_somatic_symptoms | intrepid$F3201Mild_Depressive_Episode_with_somatic_symptoms | intrepid$F322Severe_Depressive_Disorder

count(intrepid$Depression_CISR)

## 77 depressed patients##

write.csv(intrepid,"INTREPID_barts_data_CISR_cleaned_Final.csv", row.names=F, quote=F)

data = read.csv("INTREPID_barts_data_CISR_cleaned_Final.csv", header = TRUE, sep = ",", as.is=T)

### confirming reliability of data ###

library(data.table)

data <-fread("INTREPID_barts_data_CISR_cleaned_Final.csv")

### All done ###
